# Fabrication and Finite Element Analysis of Composite Elbows

**DOI:** 10.3390/ma12223778

**Published:** 2019-11-17

**Authors:** Tianlu Qiao, Guowei Zhang, Yue Xu, Boming Zhang

**Affiliations:** 1 School of Materials Science and Engineering, Beihang University, Beijing 100191, China; 2 Senior Expert Technology Center of Chinese Academy of Science, Beijing 100049, China

**Keywords:** composite tube beam, elbow, load capacity, finite element method validation

## Abstract

“Tube beams” are common lightweight structures, which have domestic and industry applications, and are often subjected to complex multidirectional loads. Therefore, metals with mature manufacturing methods and isotropic properties are commonly used in the fabrication of these structures, which are preferred to be lighter in weight. Although polymer matrix composites are generally used for weight reduction, their conventional manufacturing methods, such as pultrusion and filament-winding, cannot meet the isotropic requirements. Moreover, research on bent tube beams (elbows) is rare. Therefore, a self-made glass fiber/epoxy polyvinyl ester fabric prepreg and a self-designed mold were used in this study to prepare an isotropic composite double-bent elbow by a silicone rubber airbag-assisted process. The load capacity of the elbow was tested and validated by the finite element method. A strength and deformation of up to 3448 N and 2.84 mm respectively, were achieved. The simulation and experimental results were consistent: the error for the load capacity and deformation was only 4.15% and 7.75% respectively, under the max stress criterion.

## 1. Introduction

The main shaft of a textile machine, the anti-collision beam of a car, the missile pylon of a fighter aircraft, and a pressure gas container, are common examples of “tube beam” structures. These structures are usually required to withstand multidirectional complex loads. Therefore, metals with mature manufacturing methods and isotropic properties are commonly used in the fabrication of these structures. However, lightweighting is generally preferred in the aircraft [1], aerospace, and automotive fields [2], because reduced weight, for example, adds stability to a textile machine shaft and improves its precision. Similarly, it can increase the driving mileage of electric automobiles and improve the transportation convenience of pressure gas containers.

In recent years, fiber-reinforced thermosetting polymer matrix composites have received widespread attention in the fields of aviation, aerospace, weapons, ships, etc. [3,4], owing to their high specific strength and modulus, high-performance designability, and excellent comprehensive performance. These materials have been widely used in the aerospace industry as they can significantly reduce the weight of structures while ensuring their performance.

However, nowadays, the manufacturing methods of the high-efficiency and low-cost fiber-reinforced polymer matrix composites mainly include filament-winding and pultrusion processes: the filament-winding method is mainly performed in three directions, namely circumferential, longitudinal, and spiral winding. However, the fibers are oriented in a single direction, and therefore, cannot bear multidirectional loads. Similarly, pultrusion is only suitable for preparing structures with fixed cross-sections and unlimited lengths. Moreover, as the composites only contain axial fibers, they cannot bear multidirectional loads like the metal structures.

The quasi-isotropic properties of the glass-fiber-reinforced polymer matrix woven fabric composites [5] are similar to those of the metal in the plane because they contain bidirectional or multidirectional fibers and can carry multidirectional loads. In the meantime, the silicone rubber airbag-assisted fabrication method has gained popularity as a low-cost manufacturing technology for composite materials. This method primarily uses a silicone rubber airbag as a core mold to shape the composite material body as required. Compared with the traditional rigid molds, the manufacturing process of the silicone rubber is simple and the raw materials are sufficiently available; thus, it is less expensive. Moreover, while fabricating the complex composite structures, both curing and molding can be completed simultaneously, thereby shortening the duration of the production process. Furthermore, an increase in the pressure of the airbag can also increase the fiber volume by 20–30%, which significantly enhances the mechanical properties of the component [6]. Lehmann [7] placed the preform and the airbag in a metal mold, and pressurized the airbag, such that the preform was closely attached to the inner wall of the mold. Then, resin was injected into the preform and the mixture was allowed to solidify. Thus, a hollow tube with higher strength was obtained. Wang [8] studied the possibility of using silicone rubber as a raw material for the airbags to assist the resin transfer molding (RTM) process and reported that its tensile strength was 5 MPa, which is much higher than the internal pressure required for the airbag. Moreover, the elongation at break was 300%, indicating the high elasticity and strong deformation ability of the silicone rubber, which meets the requirements of components with complicated shapes. Moreover, the tear strength was much higher than the peel strength, and therefore, the removal of the molds was easy, which satisfies the requirements for the fabrication and demolding of the composite structures. Furthermore, Lu [9] tested the tensile, tear, and peel strengths between the silicone rubber and the testing molds after heat aging under actual application conditions of airbags, and proved that the silicone rubber airbags can be used more than 100 times. Thus, the silicon rubber airbag-assisted method is ideal for producing complicated composite materials’ structures.

Thus far, many scholars have researched the fiber-reinforced polymer matrix composite straight tube beams [10,11]. Since the 1990s, some scholars have explored the possibility of minimizing the beam weight while ensuring the performance of the straight tubes. This could be primarily achieved by using polymer matrix composites in combination with a lightweight aluminum alloy: the studies by Triantafillou [12] and Broughton [13] laid the foundation for this approach. The aluminum alloy/carbon fiber-reinforced polymer (CFRP) composite tube beam was prepared by attaching a unidirectional carbon fiber-reinforced epoxy matrix composite to the upper and lower surfaces of the hollow aluminum alloy square tube by Triantafillou. The mechanical properties of the composite tube beam were predicted by calculations, and its stiffness and strength were tested by the three-point bending method. The results showed significant improvement in the performance of the composite tube beam in comparison with the original aluminum alloy tube, while the failure mode was the buckling of the materials. These experimental results were consistent with the calculated results. Jung [14] conducted an in-depth study of the fabrication method using a silicone rubber airbag-assisted method to prepare a glass fiber-reinforced epoxy matrix composite-wrapped, aluminum alloy/glass fiber-reinforced polymer composite (GFRP) tube beam. The three-point bending method was used to evaluate the effects of the adhesive layer between the alloy and the composites. Furthermore, the influence of the thickness and angle of the composite layers on the mechanical properties and energy absorption of the composite tube beam were investigated, and the fracture characteristics of the composite tube beam were analyzed.

Furthermore, lightweighting can also be achieved by filling the tube beam with a lightweight material, which can transfer the cross-section of the beam from a ring to a circle, thereby further improving its stiffness. Eksi [15] prepared composite tube beams with reduced specific modulus from the outside to inside by externally wrapping the unidirectional GFRP or CFRP composites and internally filling them with PA6 (cast-polyamide) and PP (polypropylene). The influence of the type, thickness, and angle of the external and internal materials on the mechanical properties and energy absorption of the composite tube beams were investigated. Xiao [16] prepared composite tube beams filled with aluminum honeycombs in thin-walled CFRP matrix composite tubes, and predicted the fiber tensile and compression failure as well as matrix tensile and compression failure according to the improved Chang-Chang failure criterion [17,18]. Finally, the effects of the wall thickness, carbon fiber direction, stacking order, and impact velocity on the flexural strength of the composite tube beams were investigated. Yang [19] prepared composite honeycomb sandwich tube beams, and studied the effects of the composite layer thickness, angle, and honeycomb core height on their mechanical properties using ANSYS (12.0). The bending and torsion properties of the composite tube beams were tested and compared with those of the simulation results. Muttashar [20] fabricated multi-celled GFRP beams by using epoxy to adhere 2–4 hollow pultruded GFRP square tubes and filling the top tube by concrete. The results of static four-point bending tests indicated that the capacity as well as the stiffness of the beam are enhanced. In Mirmiran’s work [21], the structural behavior of concrete filled fiber reinforced polymer (FRP) tubes under the monotonic load and cyclic load are investigated by the finite element method. In Hangai’s work [22] and his further study [23], aluminum foam-filled thin-wall steel tube and aluminum tube were produced by friction welding.

However, to the authors’ knowledge, despite the abundant research on straight composite tube beams, research on bent composite tube beams (elbows) is rare. Chang and Springer’s research [24] laid the foundation for this area. They calculated the stresses and strains in bends made of fiber-reinforced composites and estimated the strengths of bends by a finite element method. The strength was predicted using the Tsai–Hill criterion for in plane failure and the Chang-Springer criterion for out of plane failure. Results showed the effects of geometry and ply orientation on the strength and the mode of failure. However, the bends were not bent tubes. Karama [25] simulated the static behaviors of connecting pipes made of glass fiber-reinforced thermoplastic composites under internal pressure with hydrostatic end effect and validated the results by experiments. The numerical and experimental results showed a satisfactory consistency for the different configurations tested. Abdelouahed [26] investigated the behaviors of composite structures of a tubular model connected by an elbow in the middle in Abaqus. They presented the effectiveness of the Hashin criterion under complex geometric and inner pressure loading conditions. The numerical results illustrated the mode of failure as well as the response of composite elbows. Lei and his group [27] completed the simulation of the filament-winding process of composite elbows based on the geodesic method in Matlab, and also predicted the burst pressure, stress distribution, and failure index of each part of the elbow at winding angles 60°, 70°, and 80° using the finite element method in ANSYS. He further pointed out the advantages of the geodesic method. Nevertheless, none of them studied and produced double-bent elbows.

In this study, a double-bent composite elbow was independently produced and self-made glass fiber/epoxy polyvinyl ester matrix composites and self-designed molds were used to prepare the elbow by the silicone rubber airbag-assisted manufacturing method. The load capacity of the composites was tested using the self-designed mold on a universal testing machine. Subsequently, the finite element method was used to simulate the mechanical properties of the structure, and the results were consistent with the experimental results.

## 2. Model and Material

### 2.1. Application and Model

**Figure 1:**
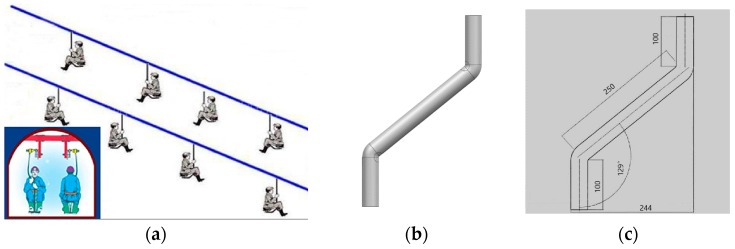
(**a**) Industrial application, (**b**) structure of the double-bent elbow, (**c**) structural parameters.

As can be seen from Figure 1a [28], this elbow is designed for the industrial application, mine ropeway. Therefore, its structure (double bends), geometry, and dimensions had already been defined. It used to be made of metal, which is available, cheap, and has mature manufacturing methods. However, it is too heavy to be delivered. Therefore, we transferred the material from metal to composite materials. Since the mine ropeway should subject to multidirectional loads, we used glass fiber-reinforced polymer matrix woven fabric composites, whose quasi-isotropic properties are similar to those of the metal in the plane. Moreover, because pultrusion and filament winding are unavailable in this case, the silicon rubber airbag-assisted manufacturing method was applied.

Figure 1b presents the structure of the double-bent elbow and Figure 1c displays its specific structural parameters (unit: mm). The elbow model comprises two short straight tubes at both ends, two corners, and a long straight tube in the middle. The arc angles of both corners are 129°, and the length of the straight tube between the two corners is 250 mm. The length of the tubes at both ends is 100 mm, and the distance between their outer edges is 244 mm. The elbow has been designed such that its outer diameter is 40 mm and wall thickness is 4 mm.

### 2.2. Material

The manufacturing method for the continuous fiber polymer matrix prepreg is generally divided into two methods: melt impregnation [29] and hot melting [30]. In the former method, the resin and the solvent are first mixed to form a solution in a prescribed ratio, followed by impregnating the fiber or fabric in the glue tank at a certain speed. Finally, the solvent is removed by heating. The hot melting method is developed based on the melt impregnation method and can be of two types: direct hot melting (one-step method) and film calendering (two-step method). In the one-step method, the resin is placed in a glue tank, melted into a low-viscosity liquid by heating, and then the fiber is allowed to successively pass through the yarn stretching machine, tension control system, glue tank, several sets of the rubber rolls, and fiber rearrangement system. Finally, it is rolled up to form a prepreg. 

**Figure 2:**
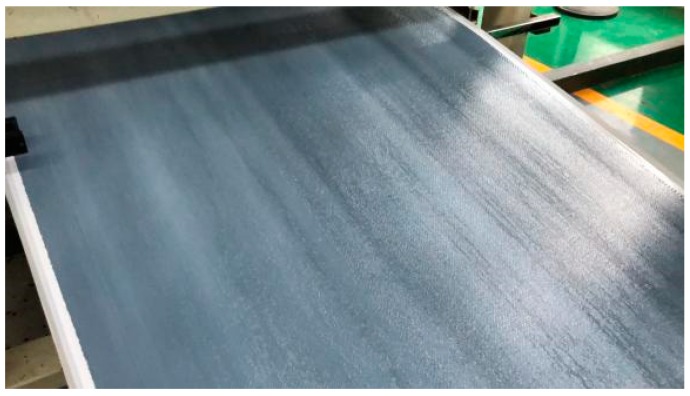
Self-made fabric prepreg.

This study uses the one-step method to prepare self-made glass fiber/epoxy polyvinyl ester prepreg (Figure 2), which can provide superior mechanical properties, corrosion resistance, and flame retardation at lower material costs, lower curing temperatures, and faster producing rates (5 m/min) and curing rates (1 min/mm). Moreover, the prepreg does not contain styrene, and has a little volatile content, which is environmentally friendly. The fabric prepreg has a single layer thickness of 0.2 mm and resin content of 45%. It is very hard to prolong the preservation time while increasing the curing rate for thermosetting resin matrix composite materials. However, this prepreg can be stored for up to one month at 25 °C and six months at −18 °C. Table 1 summarizes the mechanical properties of the prepreg after curing.

## 3. Technical Process

**Figure 3:**
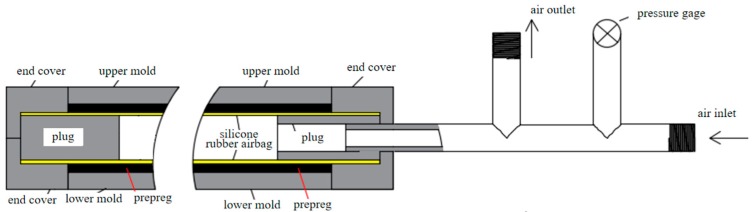
Self-designed mold.

Figure 3 shows the mold designed for this study, which comprises two plugs, upper and lower molds, and an end cover from the inside to the outside. The outer molds (including the upper and lower molds) are connected by bolts. When in use, the plug at one end is combined with the end cover to seal this end, and the plug at the other end is hollow to allow air inside. The air inlet and outlet have been designed to inflate and deflate the silicone rubber airbag, respectively. During the preparation of the double-bent elbow, the inflated silicone rubber airbag and the upper and lower molds together shape the prepreg.

**Figure 4:**
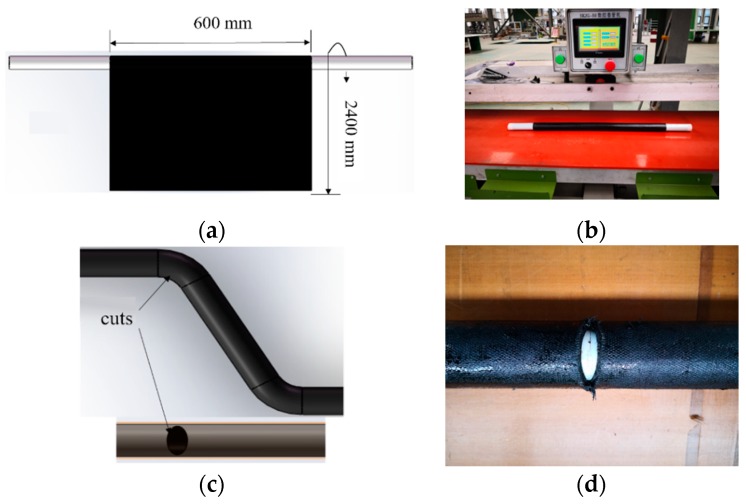
(**a**) Laying of prepreg, (**b**) the tube coiling machine, (**c**,**d**) cutting off at corners.

The following are the details of the technical process:

Firstly, the fabric prepreg was rolled up on a predetermined mold using a tube coiling machine (SKJG-50). Twenty layers were laid, ensuring no gaps between the layers (Figure 4a). The total length of the winding was 2.4 m and the length of the tube was 0.6 m. After the layering was complete, the wall thickness of the initial tube beam was approximately 4.4 mm (Figure 4b).

Secondly, the predetermined mold was removed, and the excess prepreg at the corners of the elbows was cut off to prevent the formation of wrinkles due to accumulation. For this, an elliptical cut was made at both corners, spanning half of the tube beam in the radial direction, and up to a maximum of 25 mm along the axial direction (Figure 4c,d).

**Figure 5:**
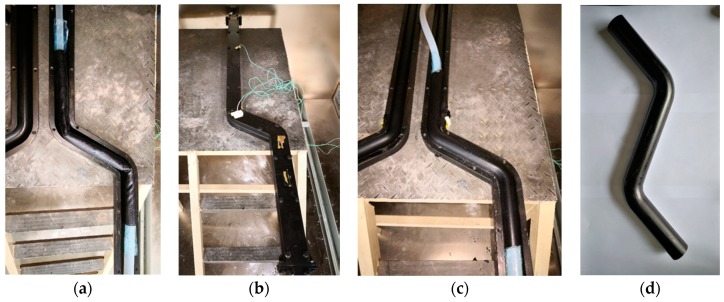
(**a**) Corner strengthened, (**b**) attached thermocouples and heating of the elbow, (**c**) demolding of the elbow, and (**d**) polished elbow.

Thirdly, the silicone rubber airbag was placed inside the prepreg (with cuts at both corners), the whole structure was placed in the lower mold that was coated with a release agent, and the silicone rubber airbag was slightly inflated to provide an initial shape. The discontinuity in the fiber due to the cutting eventually causes the corners to be the weakest parts of the elbow. Therefore, these parts are specifically strengthened by wrapping the same prepreg around the corners of the elbow, as shown in Figure 5a. Next, a plug was placed in the silicone rubber airbag. Subsequently, the entire mold was sealed with bolts (except one bolt hole, which was used to place a thermocouple for monitoring the temperature inside the mold), and a thermocouple was attached to the outer wall of the mold (Figure 5b).

Fourthly, the mold was placed in an oven, and a pressure of 0.8 MPa was applied to the airbag to ensure that the prepreg closely adheres to the outer mold. The temperature was increased at a rate of 1 °C/min, and after the air temperature and the temperature in the mold reached 150 °C, the prepreg was completely cured by maintaining this temperature for 30 min (Figure 5c).

Finally, after the curing process was complete, the temperature was lowered to 60 °C at the rate of 3 °C/min, and then air cooled to 30 °C. The elbow was demolded, the rough parts at both its ends were cut off, and the surface was polished to obtain a finished, isotropic glass fiber composite, double-bent elbow (Figure 5d).

## 4. Characterization

**Figure 6:**
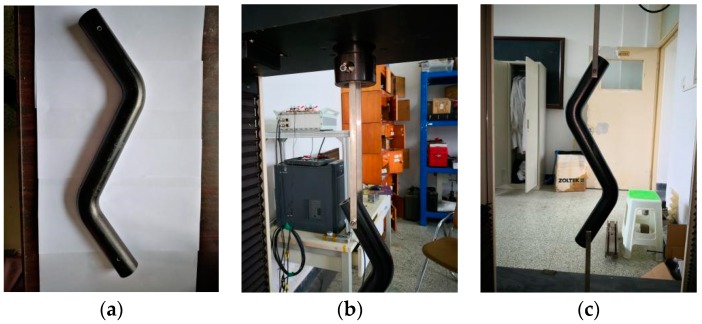
(**a**) Punched elbow, (**b**) testing molds, (**c**) load capacity testing.

As shown in Figure 6, the load capacity of the double-bent elbow was evaluated using a self-designed mold on a universal testing machine (WDW-100). As shown in Figure 6a, after the surface treatment, a hole of 5 mm diameter was punched at a distance of 10 cm from both joints of the elbow. There is no norm for experimental testing of double-bent elbows. However, the experiment consulted testing norm “ASTM D 3039”, which is “test method for tensile properties of polymer matrix composite materials”. The elbow was lifted and loaded slowly (0.05 mm/min) to ensure it is in the static state until its failure (Figure 6c). The experiment is displacement control. Moreover, the hinges are made of 40Cr, whose modulus is about 210 GPa, and their dimensions are 4 mm × 12 mm × 150 mm. When they are elastically deforming, their deformation $\Delta l\;=\;\frac{Fl}{EA}$, where *F* is Force, *l* is length, *E* is modulus, and *A* is cross-section area. Since the elbow is hung by two hinges at each end, the force of each hinge is half as much as the force of the elbow. After calculating, the deformation of the hinge is less than 10^−4^, which is negligible.

## 5. Discussion

### 5.1. Load Capacity

**Figure 7:**
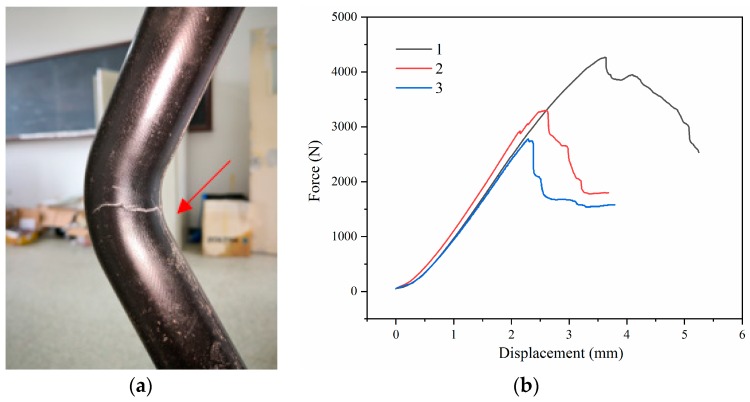
(**a**) Failure mode of the elbow, (**b**) force-displacement curves.

In this study, we prepared three elbows through the technical process mentioned and they are numbered as 1, 2, and 3. Table 2 summarizes the results of elbows 1, 2, and 3 tested using the self-designed mold on a universal testing machine. As mentioned earlier, to prevent the prepreg from accumulating and forming wrinkles, a portion of its corner was cut, which resulted in a discontinuity in the fiber. Thus, despite the subsequent reinforcement, the corner was the weakest part in the structure. The final failure mode of the elbow appears as a sudden crack at the upper corner (see Figure 7a), corresponding to the sharp fall in the force-displacement curve, as shown in Figure 7b: before the crack generation, the elbow exhibits a good load capacity, and the force rises steadily, in an almost linear manner, with the displacement. At approximately 3000 N, a crack is generated at the corner, which rapidly expands. As a result, the load capacity of the elbow is sharply reduced to less than 2000 N and continues to gradually decline. Finally, we observe that the average load capacity of the composite elbow is 3448 N, and the average displacement at failure is 2.84 mm. During the preparation of the elbow, the excess material at the corners had to be manually cut off, which causes significant discontinuity, and therefore, the variability of the load capacity of the elbow is high. The wall thicknesses were measured from the boundaries of the elbows, but the elbows failed because of the discontinuity of fibers at the corners. Thus, there is no pattern between load capacity and wall thickness.

### 5.2. Finite Element Method (FEM) Validation

**Figure 8:**
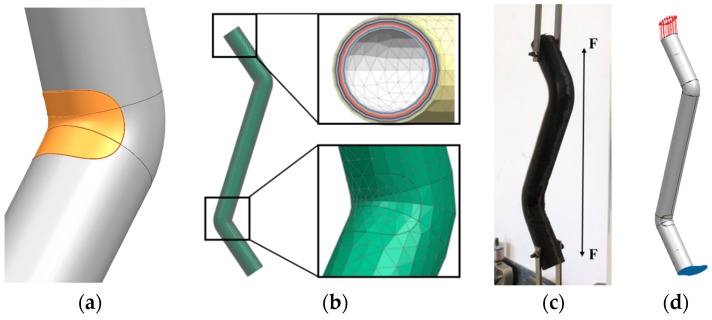
(**a**) Separate parts at the corner, (**b**) meshes and layers, (**c**) experimental conditions, and (**d**) simulated conditions.

The three-dimensional (3D) model of the elbow was imported into the finite element software, Simcenter. The model was meshed, material properties were provided, and the constraints and loads were imposed according to the actual testing conditions. Because of the discontinuity in the fibers at the cuts, there must be an area around the cuts, which is only adhered by resin. Therefore, in order to simulate the corner cuts and wrapped prepreg around the corners in the preparation process, the regions having the same size as the actual cuts were separately formed at the upper and lower corners of the finite element model (Figure 8a), and the material properties of the epoxy polyvinyl ester resin were assigned to the inner fifteen plies of the region (specific tensile modulus 3.5 GPa, strength 85 MPa). The material properties listed in Table 1 were assigned to the other areas of the elbow and the outer five plies of the cuts regions.

A two-dimensional (2D) triangle mesh “CTRIA3” was used, which is more reliable for composites simulation analysis. The mesh size at the cut was 5 mm, while the rest of the mesh was 10 mm, such that the two mesh nodes coincided (Figure 8b). Table 3 is the mesh sensitivity analysis in the Tsai–Hill criterion. As can be concluded from this table, results are not sensitive to the mesh size when the mesh is small enough and we set these parameters for time-saving. After successfully meshing the outer surface of the elbow, the mesh was stretched 20 layers inward, such that each layer was 0.2 mm thick (Figure 8b). The final finite element model contained a total of 3031 units and 1538 nodes. As shown in Figure 8c,d, we simulated the actual experimental conditions, two nodes on the bottom boundary were fixed and other nodes were pinned. A uniformly increasing load was applied on the upper end of the model, and finally, we obtained a solution until the elbow failed.

At this stage, the simulation and analysis of the composite structure failure is still under development. Especially, the analysis of the mechanical properties of the composite elbows is basically blank. Besides, the selection of the material failure criteria is often directly related to the accuracy of the ultimate strength simulation of the composite structures. Therefore, this study compared the effects of different failure criteria on the validation of the mechanical properties of the elbow, as shown in Table 4.

Among the composite strength criteria that do not distinguish failure modes, the ultimate strength criteria (including the Max-Stress criterion and the Max-Strain criterion) are used the most frequently [31]. Under ultimate strength criterion, material fails when any component of the principal stress (strain) reaches the failure stress (strain). However, it does not take the interaction into account. The Tsai–Hill criterion takes the interaction of the three principal stresses into consideration, but in principle, it only can be applied to composite materials with the same tensile and compressive properties. Based on the Tsai–Hill criterion, the stress term is introduced in the Hoffman criterion, the longitudinal and transverse tensile and compressive strengths are distinguished, and the effects of differences between tensile and compressive properties on material failure are considered. In order to improve the consistency between the theoretical and the experimental results, Tsai and Wu proposed a unified Tsai–Wu criterion in the form of tensor: in the Tsai–Wu criterion, the stress primary term can reflect the different effects of the tensile and compressive strengths on material failure, and the stress quadratic term can establish a smooth elliptical failure envelope surface [32]. 

The most commonly used failure criteria are the max stress, max strain, Tsai–Wu, Tsai–Hill, Hoffman, and Malmeister criteria. The guidelines for all except max stress and max strain criteria can be expressed as follows:

*F_i_σ_i_* + *F_ij_σ_i_σ_j_* = 1 (1)

However, the most important difference between these criteria is the calculation of the interaction term: *F*_12_. *F*_12_ is obtained by the biaxial experiment in the Tsai–Wu criterion and explicitly expressed by the unidirectional intensity in the other criteria. 

Among the above-mentioned failure criteria, the Tsai–Hill criterion is relatively simpler and convenient to use, although its accuracy is relatively inferior. Generally speaking, max stress and Tsai–Wu criteria are more accurate and appropriate for composite materials failure analysis.

The max stress criterion is expressed as follows [33]:

Assume that the tensile strength in direction 1 of the material is *X_T_*, the compressive strength is *X_C_*, the tensile strength in direction 2 is *Y_T_*, and the compressive strength is *Y_C_*. In direction 3, the tensile strength is *Z_T_*, and the compressive strength is *Z_C_*. The shear strength in direction 23 is *R*, and *S* in direction 13, *T* in direction 12. *σ*_11_ is the normal stress in direction 1, *σ*_22_ in direction 2, *σ*_33_ in direction 3. *σ*_12_ is the shear stress in direction 12, *σ*_23_ in the direction 23, *σ*_13_ in direction 13. When any of the relations in Equation (2) is satisfied, material fails.

(2) $$-X_{C}\geq \sigma_{11},\;\sigma_{11}\geq X_{T},\;-Y_{C}\geq \sigma_{22},\;\sigma_{22}\geq Y_{T},\;-Z_{C}\geq \sigma_{33},\;\sigma_{33}\geq Z_{T},\;\left|\sigma_{23}\right|\geq R,\;\;\left|\sigma_{13}\right|\geq S,\;\left|\sigma_{12}\right|\geq T$$

The Tsai–Wu criterion is expressed as follows [33]:

When the tensor relationship in Equation (3) is satisfied, material fails.

(3) $$F=\overset{6}{\underset{i=1}{\sum }}F_{i}\sigma_{i}+\overset{6}{\underset{i=1}{\sum }}\overset{6}{\underset{j=1}{\sum }}F_{ij}\sigma_{i}\sigma_{j}\geq 1$$

where $F_{1}=\frac{1}{X_{T}}-\frac{1}{X_{C}}$, $F_{2}=\frac{1}{Y_{T}}-\frac{1}{Y_{C}}$, $F_{3}=\frac{1}{Z_{T}}-\frac{1}{Z_{C}}$, $F_{11}=\frac{1}{X_{T}X_{C}}$, $F_{22}=\frac{1}{Y_{T}Y_{C}}$, $F_{33}=\frac{1}{Z_{T}Z_{C}}$, $F_{44}=\frac{1}{R^{2}}$, $F_{55}=\frac{1}{S^{2}}$, $F_{66}=\frac{1}{T^{2}}$, $F_{12}=-\frac{1}{2}\frac{1}{\sqrt{X_{T}X_{C}Y_{T}Y_{C}}}$, $F_{13}=-\frac{1}{2}\frac{1}{\sqrt{X_{T}X_{C}Z_{T}Z_{C}}}$, $F_{23}=-\frac{1}{2}\frac{1}{\sqrt{Y_{T}Y_{C}Z_{T}Z_{C}}}$. $\;\sigma_{i}$ is the principal stresses.

Table 4 presents the error between the simulation and actual experimental results under each criterion. We observe that the simulation results are in good agreement with the experimental results, and the errors of load capacity and deformation are nearly within 10%. According to the comprehensive analysis, the max stress criterion is most accurate for failure simulation of the composite elbow in this study: the prediction error of load capacity is 4.15%, while that of deformation is slightly higher, i.e., 7.75%.

**Figure 9:**
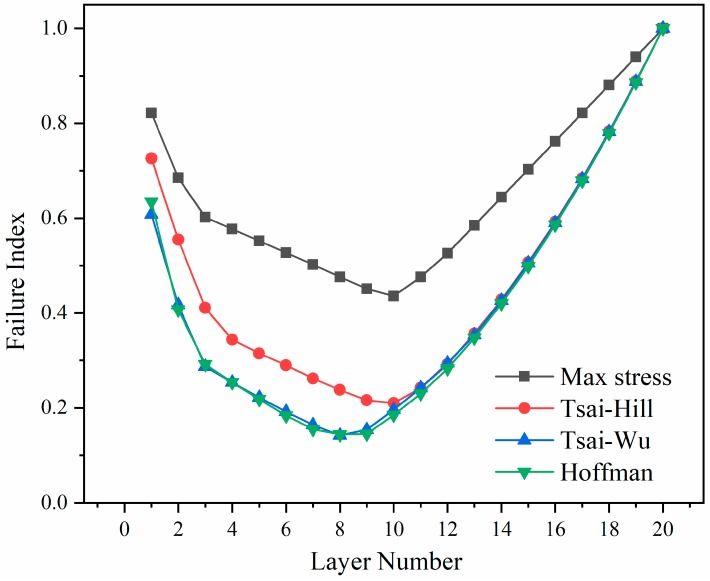
Failure index under different failure criteria.

The finite element simulation results show that the elbows did not produce interlayer failure (the interlayer failure index was less than 1). Moreover, Figure 9 shows that regardless of the failure criteria, the failure index of each layer of the elbow first increases and then decreases with the number of layers, from the inside to the outside. We further observe that the failure index reaches 1.0 at the outermost layer of the elbow, which corresponds to the sudden cracks that occur in the layer during the experiment, resulting in the overall failure of the elbow. At the same time, Figure 9 confirms that the results of load capacity in Table 4 also refer to the tensile load that the elbow can withstand when the failure index of the outermost layer of the elbow reaches 1.0.

As mentioned above, in the max stress criterion, the corner of the outermost ply failed first. The max stress in direction 1 is 70.53 MPa, the stress in direction 2 is 2.80 MPa, and the shear stress is −50.95 MPa. Because the shear strength of the material is 51 MPa, the reason why the structure cracked is that the shear strength is insufficient.

The same as the max stress criterion, in the Tsai–Wu, Tsai–Hill and Hoffman criteria, the corner of the outermost ply also firstly failed. The max stress in direction 1 and 2, and the shear stress are shown in Table 5. Interaction of the stresses is considered in all of the three criteria. The structure mainly failed due to shear stress.

**Figure 10:**
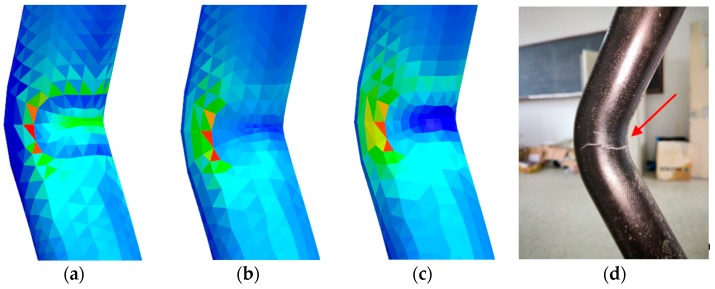
Force cloud diagram of the upper corner of the (**a**) first, (**b**) tenth, and (**c**) twentieth layer of the elbow, and (**d**) failure mode of the elbow.

Figure 10a–c show the force cloud diagrams of the upper corner of the first, tenth, and twentieth layers of the elbow from the inside toward the outside: the brighter the color, the greater the force. The figure shows that the two corners of the elbow are the weakest parts in both inner and outer layers, resulting in stress concentration in these areas. Besides, the specific force from the innermost to the outermost layer is also slightly different. The most stressed part is the corner in the innermost layer; however, the force gradually diffuses. The main force area gradually expands to the left and right sides of the corner until it reaches the outermost layer.

Figure 10c presents a magnified view of the corner of the twentieth layer of the elbow. We observe that the corner is the weakest point of the overall structure, while the transition between the two materials is still weaker. Although the corner and the left and right sides are simultaneously loaded, the force is maximized at the joint, which in turn causes cracks and damage, corresponding to the experimental results shown in Figure 10d. This proves that the simulation results are accurate and in good agreement with the experimental results.

## 6. Conclusions

(1) In this study, a self-made glass fiber/epoxy polyvinyl ester fabric prepreg and a self-designed mold were used to prepare an isotropic composite double-bent elbow by the silicone rubber airbag-assisted manufacturing method. The load capacity was tested using a self-designed mold on the universal testing machine. The results showed that the average maximum load of the elbow can reach 3448 N, while the corresponding maximum deformation is 2.84 mm. However, during the preparation of the elbow, the excess material at the corners had to be manually cut off, which causes significant discontinuity, and therefore, the variability of the load capacity of the elbow is high. Moreover, after the preparation is complete, the silicone rubber airbag needs to be manually demolded, which considerably lowers the production efficiency of the structure.
(2) The finite element method was used to validate the mechanical properties of the overall elbow structure, and the results showed that from the innermost to the outermost layer, the stressed areas of the elbow gradually spread from the corner to its left and right sides. Furthermore, the failure mode of the simulation results is consistent with that of the actual experiment: the layer failure index first increases and then decreases from the inside to the outside, reaching 1.0 at the outermost layer. Because the failure criterion has significant influence on the structural property simulation, this study compares the error between the simulated and experimental values under different failure criteria. The results showed that the error of the load capacity is less than 10%, while the error of the deformation is about 10% under the four different criteria. Among these, the max stress criterion exhibited the highest accuracy: the simulation value of the load capacity was 3591 N, with an error of 4.15%, while the predicted value of the deformation was 3.06 mm, with an error of 7.75%.

## Figures and Tables

**Table 1:** Mechanical properties of the prepreg.

Type	Tensile Strength /MPa	Tensile Modulus /GPa	Compressive Strength /MPa	Compressive Modulus /GPa	Flexural Strength /MPa	Flexural Modulus /GPa	In-plane Shear Strength /MPa	Interlaminar Shear Strength /MPa
V2550A	293	23	193	27	282	31	51	36

**Table 2:** Tensile testing results of the elbow.

Sample Number	Load Capacity/N	Deformation/mm	Wall Thickness/mm	Outer Diameter/mm
1	4266	3.62	3.80	40.00
2	3300	2.60	4.00	39.80
3	2777	2.29	4.10	39.80
Average	3448	2.84	3.97	39.87

**Table 3:** Mesh sensitivity analysis.

Mesh Size/mm	1	2	3	4	5	7	10	15
Load Capacity/N	3208	3244	3292	3273	3235	3309	3324	3332
Deformation/mm	2.55	2.59	2.61	2.68	2.57	2.75	2.90	2.94

**Table 4:** Simulation results under different failure criteria.

Parameters	Experimental Results	Tsai–Hill	Tsai–Wu	Max Stress	Hoffman
Load capacity/N	3448	3235	3274	3591	3665
Error/%	–	6.18	5.05	4.15	6.29
Deformation/mm	2.84	2.57	2.59	3.06	3.14
Error/%	–	9.51	8.80	7.75	10.56

**Table 5:** Stress results under different failure criteria.

Stress/MPa	Tsai–Hill	Tsai–Wu	Max Stress	Hoffman
Direction 1	68.60	72.05	70.53	72.16
Direction 2	2.73	2.86	2.80	2.87
Shear Stress	−49.60	−52.04	−50.95	−52.13

## References

[1] Soutis C. (2005). Fibre reinforced composites in aircraft construction. Prog. Aerosp. Sci..

[2] Chung D.D.L. (2017). Processing-structure-property relationships of continuous carbon fiber polymer-matrix composites. Mater. Sci. Eng. R..

[3] Soliman E., Kandil U., Taha M.R. (2014). Improved Strength and Toughness of Carbon Woven Fabric Composites with Functionalized MWCNTs. Materials.

[4] Bussetta P., Correia N. (2018). Numerical forming of continuous fibre reinforced composite material: A review. Compos. Part A Appl. Sci. Manuf..

[5] Chen L., Chen D., Rong Z.J. (2018). Research progress of reinforced fabric of composite blade for turbo engine. J. Tianjin Polytech. Univ..

[6] Wei B., Zhou J.T., Yan Z.J. (2018). Development Status and Application Prospect of RTM and Its’ Derived Technologies. Guangzhou Chem..

[7] Lehmann U., Michaeli W. (1998). Cores lead to an automated production of hollow composite parts in resin transfer moulding. Compos. Part A Appl. Sci. Manuf..

[8] Wang R.N., Wu X.Q., Lou X.J. (2007). Application of Silicon Rubber Inflatable Bag in RTM Technology. Fiber Compos..

[9] Lu J.J., Wang G.Y., Zhang L. (2006). Service-Span Estimate for Silicon Rubber Inflatable Mandrel Employed in Composites Molding. Aerosp. Mater. Technol..

[10] Manoj Prabhakar M., Rajini N., Ayrilmis N. (2019). An overview of burst, buckling, durability and corrosion analysis of lightweight FRP composite pipes and their applicability. Compos. Struct..

[11] Al-saadi A.U., Aravinthan T., Lokuge W. (2018). Structural applications of fibre reinforced polymer (FRP) composite tubes: A review of columns members. Compos. Struct..

[12] Triantafillou T.C., Kim P., Meier U. (1991). Optimization of hybrid aluminum/cfrp box beams. Int. J. Mech. Sci..

[13] Broughton J.G., Beevers A., Hutchinson A.R. (1997). Carbon-fibre-reinforced plastic (CFRP) strengthening of aluminium extrusions. Int. J. Adhes. Adhes..

[14] Jung D.W., Kim H.J., Choi N.S. (2009). Aluminum-GFRP hybrid square tube beam reinforced by a thin composite skin layer. Compos. Part A Appl. Sci. Manuf..

[15] Eksi S., Genel K. (2013). Bending response of hybrid composite tubular beams. Thin Walled Struct..

[16] Xiao Y., Hu Y.F., Zhang J.G. (2018). Dynamic bending responses of CFRP thin-walled square beams filled with aluminum honeycomb. Thin Walled Struct..

[17] Tan S.C., Perez J. (1993). Progressive Failure of Laminated Composites with a Hole under Compressive Loading. J. Reinf. Plast. Compos..

[18] Hou J.P., Petrinic N., Ruiz C. (2001). A delamination criterion for laminated composites under low-velocity impact. Compos. Sci. Technol..

[19] Yang Y. (2012). Design, Manufacturing and Mechanical Properties of Composites Honeycomb Sandwich Pipes. Master’s Thesis.

[20] Muttashar M., Manalo A., Karunasena W. (2017). Flexural behaviour of multi-celled GFRP composite beams with concrete infill: Experiment and theoretical analysis. Compos. Struct..

[21] Mirmiran A., Zagers K., Yuan W. (2000). Nonlinear finite element modeling of concrete confined by fiber composites. Finite Elem. Anal. Des..

[22] Hangai Y., Saito M., Utsunomiya T. (2014). Fabrication of Aluminum Foam-Filled Thin-Wall Steel Tube by Friction Welding and Its Compression Properties. Materials.

[23] Hangai Y., Nakano Y., Koyama S. (2015). Fabrication of Aluminum Tubes Filled with Aluminum Alloy Foam by Friction Welding. Materials.

[24] Chang F.-K., Springer G.S. (1986). The Strengths of Fiber Reinforced Composite Bends. J. Compos. Mater..

[25] Karama M., Mistou S. (2013). Optimizing Textile-Reinforced Composites for Pipe Connection. Adv. Mater. Res..

[26] Abdelouahed E., Mokhtari M., Benzaama H. (2019). Finite Element Analysis of the thermo-Mechanical Behavior of composite Pipe Elbows under Bending and Pressure loading. Frattura Integrità Strutturale.

[27] Zhang B., Xu H., Zu L. (2018). Design of filament-wound composite elbows based on non-geodesic trajectories. Compos. Struct..

[28] http://www.zk71.com/products/u383402/undo487149_16142744.html.

[29] Steggall-Murphy C., Simacek P., Advani S.G. (2010). A model for thermoplastic melt impregnation of fiber bundles during consolidation of powder-impregnated continuous fiber composites. Compos. Part A Appl. Sci. Manuf..

[30] Hayes B.S., Seferis J.C. (1997). The effect of fabric tension and the number of impregnation rollers on woven fabric prepreg quality and cured laminates. Compos. Part A Appl. Sci. Manuf..

[31] Wu Y.T., Yao W.X., Shen H.J. (2015). Prediction ability analysis of macroscopic strength criteria for composites. Acta Mater. Compos. Sin..

[32] Gol’denblat I.I., Kopnov V.A. (1971). General theory of criteria of strength for isotropic and anisotropic materials. Strength Mater..

[33] Smith C.S. (1990). Design of Marine Structures in Composite Materials.

